# Adaptive carbon export response to warming in the Sargasso Sea

**DOI:** 10.1038/s41467-022-28842-3

**Published:** 2022-03-08

**Authors:** Michael W. Lomas, Nicholas R. Bates, Rodney J. Johnson, Deborah K. Steinberg, Tatsuro Tanioka

**Affiliations:** 1 Bigelow Laboratory for Ocean Sciences, East Boothbay, ME USA; 2 Bermuda Institute for Ocean Sciences, St. Georges, Bermuda; 3 Department of Ocean and Earth Science, University of Southampton, Southampton, UK; 4 Virginia Institute of Marine Science, William & Mary, Gloucester Pt., Virginia, VA USA; 5 Department of Earth System Science, University of California, Irvine, CA USA

**Keywords:** Marine chemistry, Carbon cycle

## Abstract

Ocean ecosystem models predict that warming and increased surface ocean stratification will trigger a series of ecosystem events, reducing the biological export of particulate carbon to the ocean interior. We present a nearly three-decade time series from the open ocean that documents a biological response to ocean warming and nutrient reductions wherein particulate carbon export is maintained, counter to expectations. Carbon export is maintained through a combination of phytoplankton community change to favor cyanobacteria with high cellular carbon-to-phosphorus ratios and enhanced shallow phosphorus recycling leading to increased nutrient use efficiency. These results suggest that surface ocean ecosystems may be more responsive and adapt more rapidly to changes in the hydrographic system than is currently envisioned in earth ecosystem models, with positive consequences for ocean carbon uptake.

## Abstract (web summary)

The ability of the ocean’s biota to sequester carbon is thought to be negatively affected by climate change. Here the authors use time-series data in the Sargasso Sea to show that biotic processes can buffer against these negative impacts.

## Introduction

Phytoplankton play a central role in regulating global ocean biogeochemical cycles and production in marine food webs^1–4^. Concerns about the impact of upper-ocean warming and increased stratification on phytoplankton production and carbon export have grown during the last decade^5–7^, with observations of synchronous increases in surface ocean temperature and an apparent global decrease of phytoplankton biomass, inferred from changes in chlorophyll *a* (Chl *a*) concentration^3,4^. Upper-ocean warming is thought to positively influence phytoplankton primary production by impacting their photosynthetic metabolism^8^, and has a negative effect by increasing stratification leading to reductions in vertical nutrient inputs and subsequent nutrient limitation^9^. Warming of the upper ocean is also predicted to reduce the magnitude of biological carbon export to the ocean interior by reducing upwelling of nutrients and/or by shifts in phytoplankton community composition toward smaller, less dense picophytoplankton^7,10,11^.

The magnitude of this predicted series of changes in marine biogeochemistry is in part dependent upon the stoichiometric relationship between the major macronutrients carbon (C), nitrogen (N), and phosphorus (P), with the canonical Redfield ratio set at 106 C:16 N:1 P^12,13^. Fixed stoichiometric models suggest that global net primary production (NPP) may decline by up to 20% (ref. ^5^), whereas flexible stoichiometric models indicate a < 10% reduction in NPP^7^. The Arctic Ocean and subtropical gyres appear the most sensitive global ocean regions in both models, with decreases in NPP exceeding the global mean from the models. These NPP reductions, in the models, appear to be driven by a greater decline in phytoplankton growth rate than in phytoplankton biomass stocks, supporting the general view of bottom-up (i.e., nutrient, temperature, light) controls on NPP. As with NPP, flexible stoichiometric models predict smaller decreases in the magnitude of the biological carbon pump due to modeled increases in nutrient use efficiency associated with the flexible stoichiometry of phytoplankton^14^. In terms of reducing the impact of nutrient limitation on carbon export, the benefits of flexible stoichiometry are partially negated by the reduction in mean phytoplankton cell size, which, in the models, also limits the export of carbon associated with smaller phytoplankton. Resolution of these competing processes impacting phytoplankton-mediated processes based upon in situ data is lacking. For example, in the Sargasso Sea, the resident phytoplankton community has a highly flexible macronutrient stoichiometry^15,16^. The cyanobacteria genera *Synechococcus* and *Prochlorococcus* contribute substantially to particulate carbon export^17^, resulting in an overall efficient carbon export system^18^.

In the Sargasso Sea at the Bermuda Atlantic Time-series Study (BATS) site, we have quantified relationships between phytoplankton production, nutrients, ocean physics, and carbon export for three decades. Recent studies have confirmed an inverse relationship between temperature and NPP^19^. Here, we continue to analyze long-term patterns in the coupling of NPP, carbon export, phytoplankton composition, and stoichiometry. We ask the following questions: (1) Does surface ocean warming covary inversely with nutrient inventories? (2) Do planktonic communities display a stable or variable taxonomic and elemental composition? and, (3) How do these interactions impact observed carbon export?

## Results and discussion

### Trends in hydrography

**Fig. 1:**
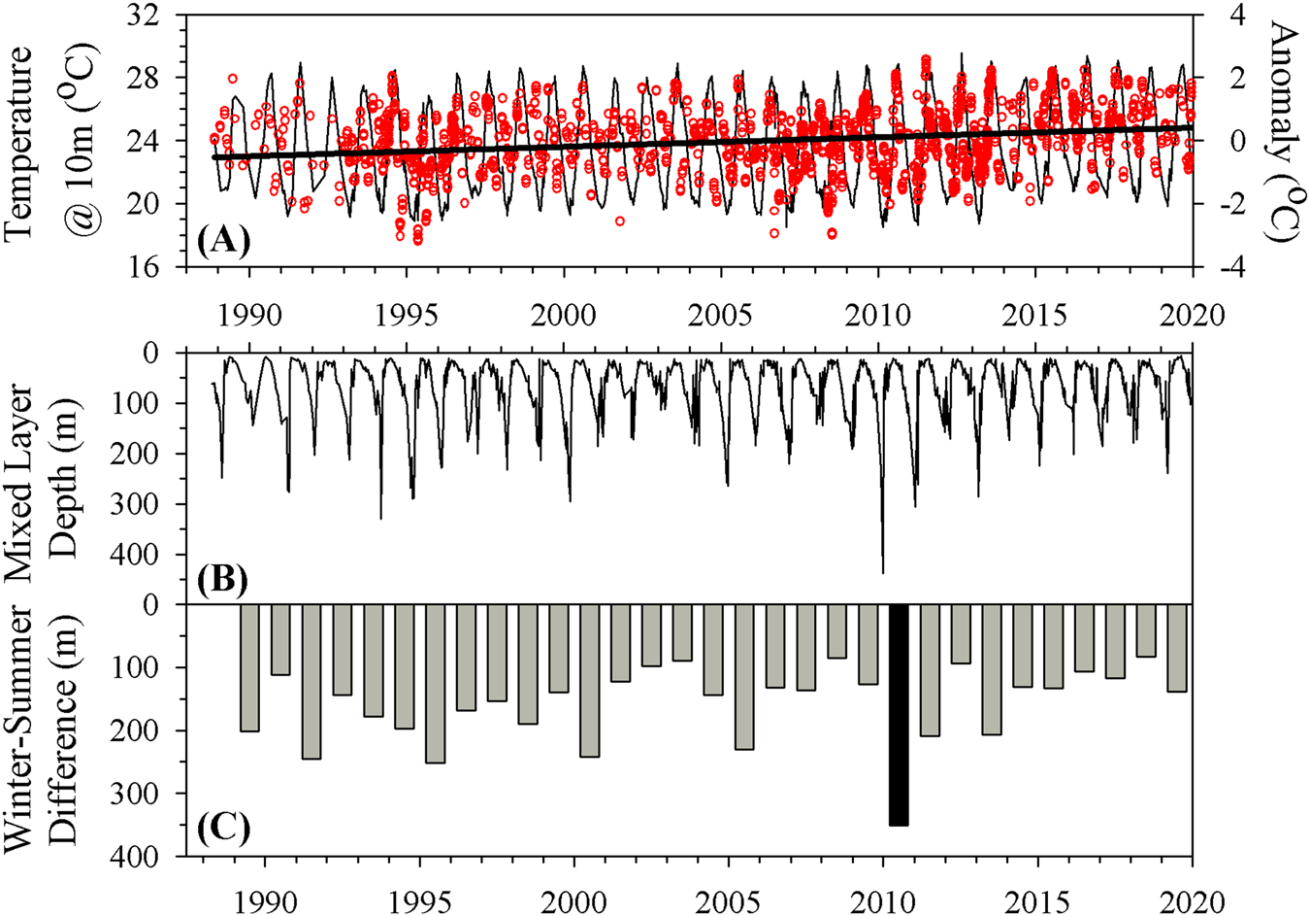
Time series of surface ocean temperature (@ 10 m), mixed layer depth, and seasonal mixing amplitude. **A** CTD temperature recorded at 10 ± 2 m (thin black line), and seasonally detrended anomaly (red circles). The thick line is the Model 1 linear regression to the anomaly data. **B** Mixed layer depths estimated from each CTD cast in the BATS record using the 0.2 ^o^C variable sigma—θ criterion^65^. **C** Difference between average maximum winter/spring (February/March) and minimum summer (July/August) mixed layer depths for each year. Note: 2010 is highlighted in a different color for reasons discussed in the text.

Surface ocean temperatures significantly increased by ~0.9 °C over the entire duration of the BATS record analyzed here (1990–2020); however, the increase has not been uniform over time^19,20^. The 1990s were characterized by a weak but significant increase in temperature at 10 m (*R*^2^ = 0.01, *P* = 0.007, 0.035 ± 0.013 ^o^C year^−1^), whereas the following decade (2000s) did not exhibit a significant temperature trend (*R*^2^ = 0.00, *P* = 0.493). The increase in near-surface temperature during the 2010s was fourfold greater than the 1990s (*R*^2^ = 0.10, *P* < 0.001, 0.119 ± 0.008 °C year^−1^) and accounted for the majority of the total temperature increase observed from 1990 to 2020 (Fig. 1A). This pattern of accelerated warming rates in the most recent decade has been previously shown^20^. Furthermore, D’Alelio et al.^19^, while examining the BATS dataset through 2015, also showed a significant decade-scale shift from no significant temperature change to significant warming beginning in ~2008, consistent with our findings. The goal of this current analysis is not to determine precisely when the period of rapid warming began, but rather to compare the biogeochemical and ecological responses during this period of rapid warming (2010s) with a period of weak to no warming (1990–2000s).

This pattern of rapidly increasing temperature through the 2010s has been shown to increase the stability of the upper ocean and reduce the formation of nutrient-rich subtropical mode water that lies below the euphotic zone^21^, which, ultimately, negatively impacts subsequent vertical nutrient inputs. During the 2010s, but not prior decades, the difference between winter maximum and summer minimum mixed layer depths significantly decreased (Fig. 1B, C; *R*^2^ = 0.46, *P* = 0.03) due primarily to shallowing of the winter maximum. This pattern is consistent with the temporal pattern in the magnitude of the wintertime North Atlantic Oscillation (NAO) index (Supplementary Fig. 1), which correlates with a range of hydrographic, chemical, and biological parameters at BATS^22,23^, and is inversely related to the depth of water column mixing during the winter period.

**Fig. 2:**
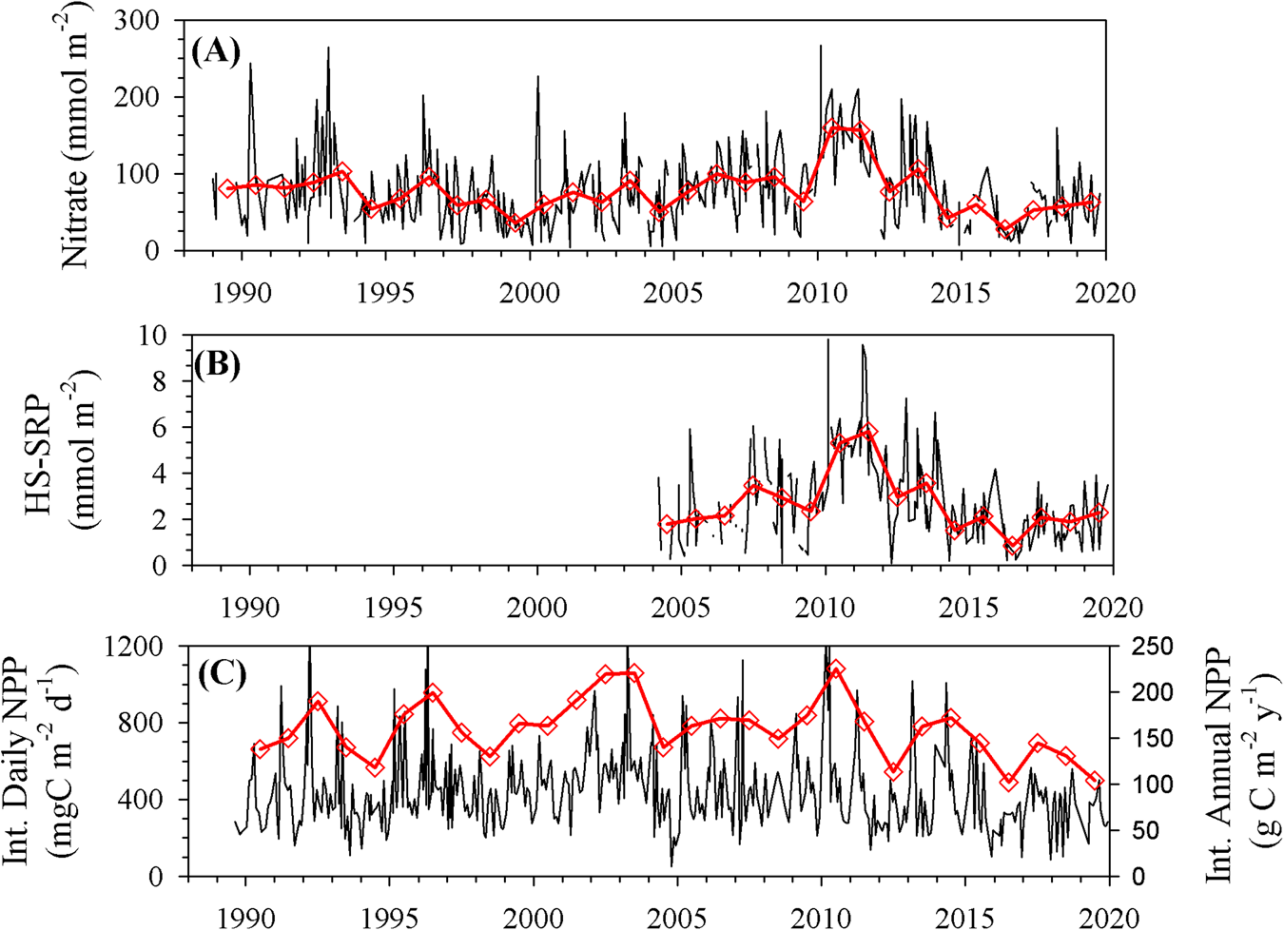
Time series of surface ocean macronutrient inventories and integrated daily and annual net primary production (NPP). **A** Nitrate and **B** high-sensitivity phosphate nutrient inventories (0–140 m; mmol m^−2^), and **C** integrated (0–140 m) daily and annual NPP. Black lines in each panel are individual cruise values, while the red lines and open diamonds are annual values. Note, annual NPP is on a different scale (right axis).

The increase in temperature and decrease in the amplitude of seasonal mixing through the 2010s was correlated with a significant net decline in inventories (0–140 m) of nitrate (Model 1 Regression, *P* < 0.001, *R*^2^ = 0.34, slope = −11.6 ± 1.6 mmol m^−2^ year^−1^) and phosphate (Model 1 Regression, *P* < 0.001, *R*^2^ = 0.36, slope = −0.43 ± 0.06 mmol m^−2^ year^−1^) (Fig. 2A, B). By the middle of the 2010 decade, nutrient inventories were reduced by 25–30% relative to their pre-2010 inventories (during which time there were no significant changes). The high-sensitivity phosphate record only goes back to 2004, but the standard autoanalyzer phosphate measurements collected at BATS also show no significant trend in inventories between 1990 and 2010 (see ref. ^24^). It is noteworthy that the anomalous 2010 wintertime NAO index^25^ resulted in a significant increase in nutrient inventories from which this decade-scale decline began. Throughout the 2010s, the dissolved organic phosphorus (DOP) inventory also decreased significantly (Supplementary Information and Supplementary Fig. 2). As predicted by global models^5^, the observed increase in surface temperature and reduction in nutrient inventories throughout the 2010s are correlated with a decade-long significant reduction in net primary production (~30%; NPP; Fig. 2C; Model 1 regression, *P* = 0.002, *R*^2^ = 0.09, slope = −22.2 ± 6.8 mg m^−2^ year^−1^).

### Trends in carbon fluxes, phytoplankton community and elemental stoichiometry

**Fig. 3:**
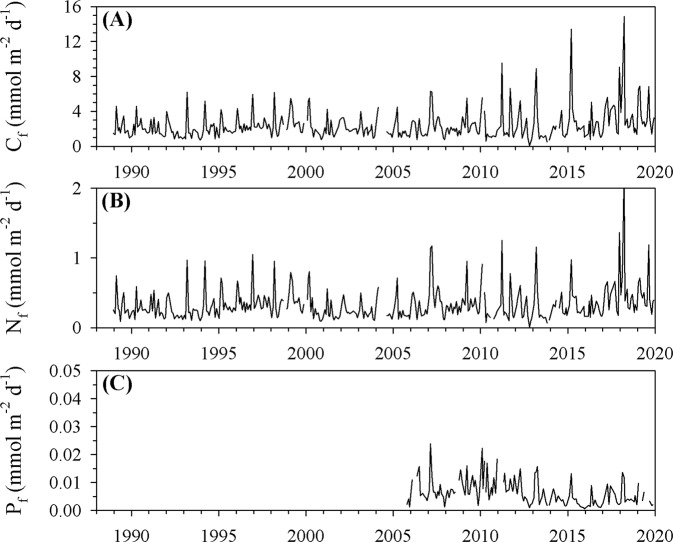
Time series of vertical elemental fluxes at 150 m. **A** Particulate organic carbon (C_f_) flux; **B** particulate organic nitrogen (N_f_) flux; **C** particulate phosphorus (P_f_) flux.

While global biogeochemical models predict this coordinated series of processes—ocean warming, increasing stratification, reduction in nutrient inputs, and subsequent decrease in NPP–they also predict a decrease in carbon export, thus reducing the magnitude of the biological carbon pump^26,27^. However, at BATS, counter to predictions, carbon export fluxes at 150 m did not significantly decrease throughout the 2010s (Model 1 Regression, *P* = 0.785) (Fig. 3A, B). While it is interesting that the increased nutrient inventory and NPP in 2010, associated with the anomalous winter NAO negative index, did not result in a significant increase in carbon export, there are several possible explanations for decoupling of carbon export and NPP in response to episodic events^28^. Despite decreasing nutrient inventories and NPP, the lack of significant decrease in carbon export suggests that these ecosystem processes became decoupled throughout the 2010s. Furthermore, the export ratio (carbon export:NPP) increased from 0.04 in 2010 to 0.16 in 2019, indicating that the ecosystem became more efficient at exporting carbon from the euphotic zone, despite the decade-long decrease in nutrient inventories and NPP.

**Fig. 4:**
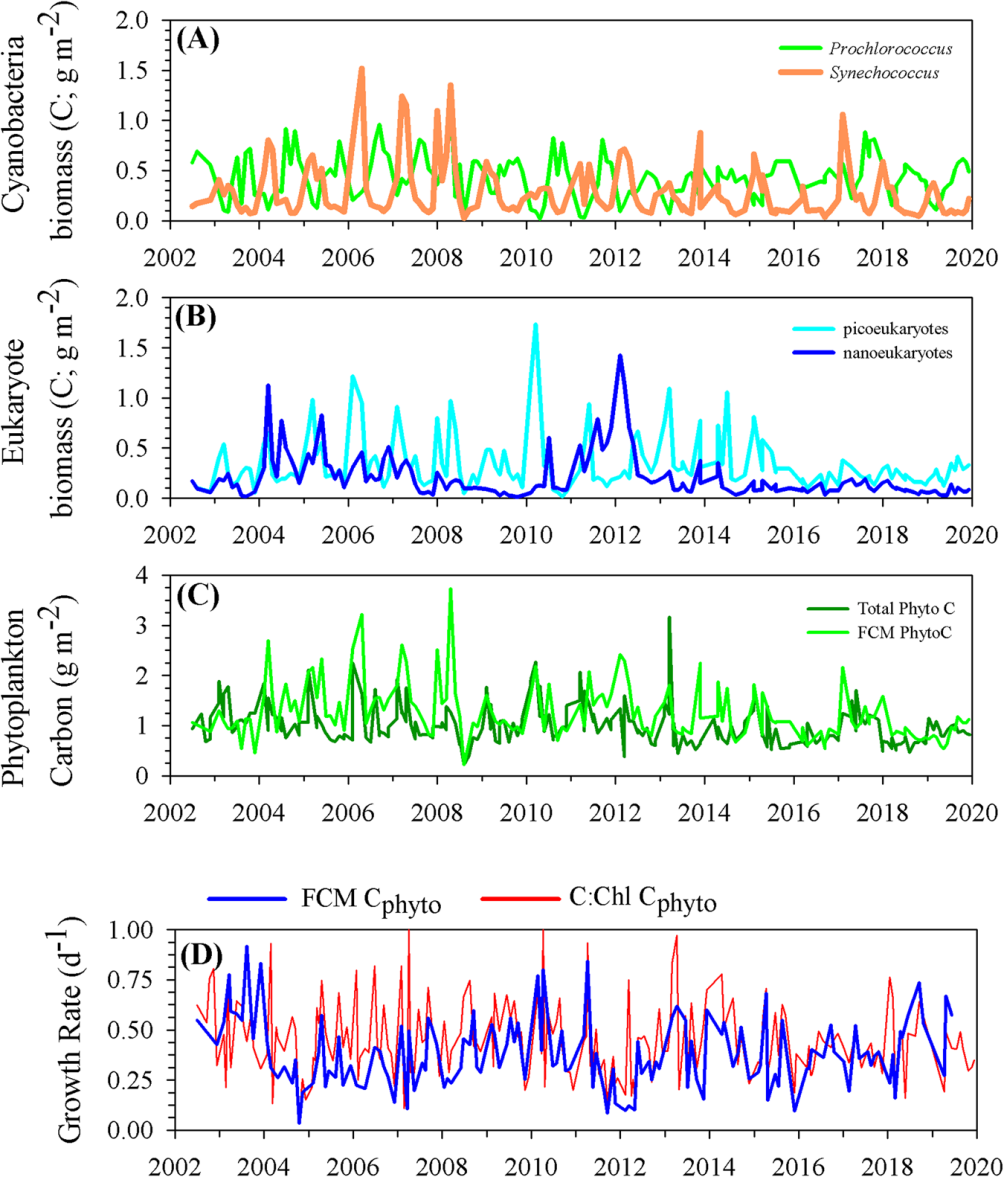
Time series of flow cytometry-derived, euphotic zone integrated phytoplankton carbon biomass (g m^−2^) and phytoplankton growth rates. **A** Biomass of cyanobacteria *Prochlorococcus* and *Synechococcus*; **B** biomass of picoeukaryotes and nanoeukaryotes; **C** total phytoplankton carbon biomass derived from the product of bulk POC:Chl slope and integrated Chl, and summed flow cytometry-derived carbon biomass estimates. **D** Phytoplankton growth rates estimated by dividing integrated in situ primary production by the two estimates of phytoplankton carbon biomass as described in (**C**).

The decline in NPP throughout the 2010s was concurrent with a reorganization of the phytoplankton community (Fig. 4). Throughout the 2010s, the distinct seasonal peak in eukaryotic biomass diminished, and their biomass remained at a baseline level statistically similar to minimum values before 2010 (Fig. 4B). Throughout the 2010s, there were no contemporaneous changes in either seasonal biomass peaks or concentrations for prokaryotic *Prochlorococcus* and *Synechococcus* biomass (Fig. 4A). The decrease in eukaryote biomass resulted in a significant decrease in summed phytoplankton biomass throughout the 2010 decade (FCM PhytoC, *P* < 0.001, slope = −0.08 ± 0.2 g C m^−2^ year^−1^; Total PhytoC, *P* < 0.001, slope = −0.05 ± 0.01 g C m^−2^ year^−1^). We were unable to detect changes in the biomass of larger, less abundant microphytoplankton, as the summed prokaryote and small eukaryote carbon biomass estimates by FCM were only marginally greater than the independently estimated total phytoplankton carbon (ANOVA, FCM PhytoC vs. Total PhytoC pre-2010, *P* = 0.08; FCM PhytoC vs. Total PhytoC post-2010, *P* = 0.04) (Fig. 4C). In addition to changes in phytoplankton biomass, we examined changes in phytoplankton growth rate. Phytoplankton growth rates, derived by separately dividing integrated NPP by FCM PhytoC and Total PhytoC, did not show significant trends, increases or decreases, in any decade (Model 1 regression, *P* > 0.2 in all comparisons) (Fig. 4D). Furthermore, mean growth rates pre- and post-2010 were not significantly different (Student’s *t* test, *P* > 0.4) from each other. Thus, the ~40% decline in NPP mentioned previously appears due primarily to the reduction in phytoplankton biomass during the 2010s, not a change in physiological condition. This change in the relative abundance of phytoplankton populations, and subsequent reduction in effective mean cell size of the phytoplankton population with the decrease of larger eukaryotes, only further confounds the decoupling of nutrients, NPP, and carbon export fluxes. Thus, explanations for the decoupling of nutrients, NPP, and carbon export likely involve changes in both phytoplankton processes (e.g., variable stoichiometry) and ecosystem processes (e.g., trophic interactions).

### Decoupling of carbon and nutrient cycles and mechanisms to maintain carbon export

**Fig. 5:**
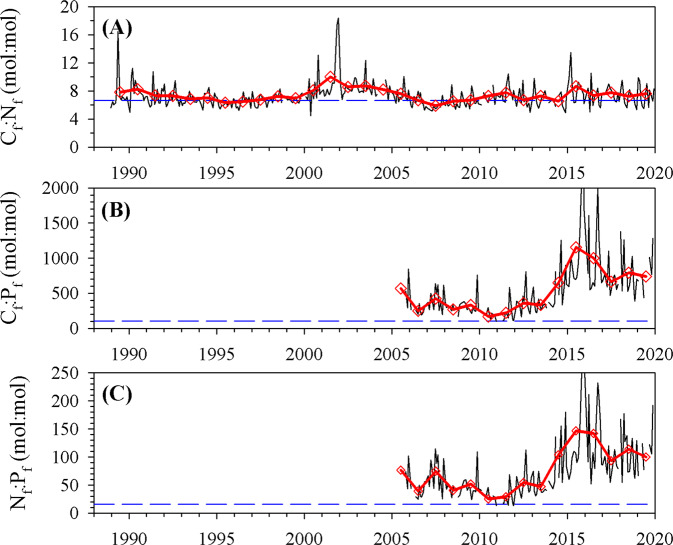
Time series of elemental flux stoichiometric ratios at 150 m. **A** Particulate C_f_:N_f_ flux ratio (thin black line); **B** particulate C_f_:P_f_ flux ratio; and **C** particulate N_f_:P_f_ flux ratio. Red triangles represent the annual mean flux ratio, and the blue dashed line represents the Redfield Ratio.

The Sargasso Sea is one of several western ocean gyre regions characterized by extremely low phosphate concentrations^29^, and is generally viewed as a phosphorus-limited system^30–32^. As such, changes in C:P stoichiometry are commonly studied in this region. Flexible macronutrient stoichiometry, associated with changes in phytoplankton community composition, has been hypothesized to buffer reductions in carbon export under conditions of increased stratification and nutrient depletion^14^. Based upon Tanioka and Matsumoto’s sensitivity ratio^14^ and the observed decrease in phosphate inventory of ~25–30%, we would expect a reduction in carbon export of ~18–22%. Throughout the 2010s, when nutrient inventories and NPP were decreasing, there were significant and concurrent changes in the macronutrient stoichiometric ratios of exported material (Fig. 5). Declining phosphorus export fluxes throughout the 2010s (Fig. 3C), without decreases in carbon and nitrogen fluxes, resulted in a significant increasing trend in C:P (Model 1 regression, *P* < 0.001, slope = 86 ± 13 units year^−1^) and N:P ratios (Model 1 regression, *P* < 0.001, slope = 11.4 ± 1.8 units year^−1^). This trend in increasing C:P and N:P ratios led to a nearly fourfold increase in the C:P (205 ± 108 versus 722 ± 226) and N:P (29 ± 14 versus 101 ± 35) stoichiometric ratios of exported material by the end of the 2010s when compared to the pre-2010 period (Fig. 5B, C), both of which were significant (Student’s *t* test, *P* < 0.001 for both comparisons). A nutrient inversion model yielded a similar C:P ratio of exported material (355 ± 65) in the subtropical North Atlantic Ocean^33^ but did not resolve temporal changes in the ratio. As both carbon and nitrogen export flux rates did not decrease, the C:N stoichiometric ratio in exported material at 150 m depth did not significantly change between the period before and after 2010 and averaged 7.23 ± 1.61 (Fig. 5A). This temporal pattern in export flux rates and all stoichiometric ratios was also observed at the 200 m and 300 m sediment trap depths (Supplementary Figs. 2 and 3). However, the change in absolute rates decreased with depth due to organic matter remineralization^34^. The increases in C:P and N:P stoichiometric ratios of exported particulate material could be due to changes in the stoichiometry of the source material (i.e., suspended particulate matter, in particular, phytoplankton), and/or due to enhanced shallow remineralization of P between the base of the euphotic zone (100 m) and the shallow sediment traps (150 m), resulting in nutrient trapping^35^. This nutrient trapping effect is not 100% efficient, as the integrated nutrient inventories do continue to decline. However, we hypothesize that this effect slows down the rate of nutrient inventory decline and thus serves as another “biological buffer” to the negative impacts of upper-ocean stratification associated with climate change.

**Fig. 6:**
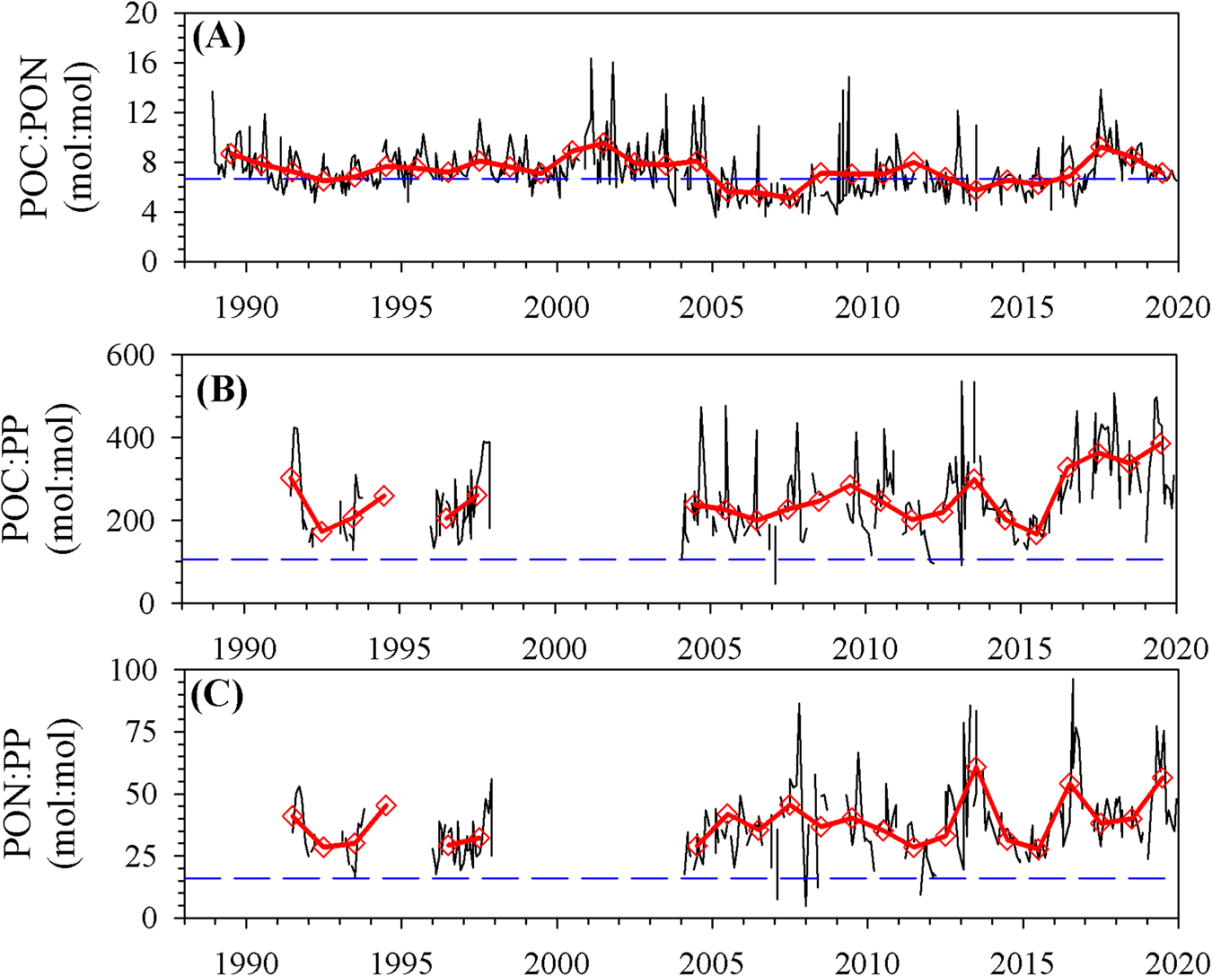
Time series of seston elemental stoichiometric ratios in the upper (0–60 m) euphotic zone layer. **A** POC:PON; **B** POC:PP; **C** PON:PP. Red triangles represent the annual mean stoichiometric ratio, and the blue dashed line represents the Redfield Ratio.

Stoichiometric ratios of sestonic particulate matter in the upper euphotic zone (0–60 m) displayed temporal patterns qualitatively similar to the same ratios in exported flux ratios (Fig. 6). C:P ratios in suspended particulate organic matter increased significantly (Model 1 Regression, *R*^2^ = 0.17, *P* < 0.001, slope = 14.5 ± 2.9 units year^−1^) throughout the 2010s and reached values higher than in the 2000s. The N:P ratio, constrained by both elements potentially being limited, also increased significantly (Model 1 Regression, *R*^2^ = 0.08, *P* = 0.02, slope = 1.48 ± 0.5 units year^−1^). Stoichiometric ratios in the lower euphotic zone (60–100 m; Supplementary Fig. 4) also mirrored temporal patterns in the upper euphotic zone but were lower in magnitude. C:P ratios in the lower euphotic zone were ~17% lower than in the upper euphotic zone, while N:P ratios were ~30% less, supporting the notion that the lower euphotic zone may be less nutrient stressed due to proximity to diapycnal nutrient inputs that are efficiently assimilated^36^. The seston pool comprises many particle types, including heterotrophic microbes, phytoplankton, microzooplankton, and detrital particles. Phytoplankton comprise a significant fraction of the total POC pool, ranging from 0.11 to 0.71 (0.30 ± 0.11; Supplementary Fig. 5). Thus, we posit that at least a portion of the increase in seston C:P ratios is linked to the shift towards increasing dominance of the phytoplankton community by cyanobacteria (Fig. 4), which are well documented to have C:P ratios in natural populations significantly greater than co-occurring eukaryotes^15,16,37^.

**Fig. 7:**
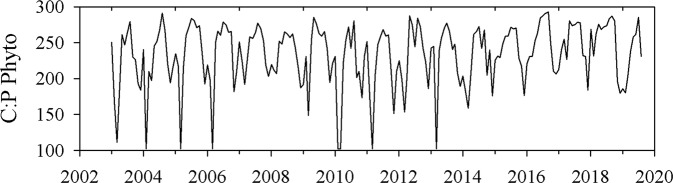
Time series of phytoplankton-specific C:P ratios calculated from the trait-based model approach of Tanioka et al.^38^. Model phytoplankton C:P in the surface 100 m is computed as a function of growth rate and POC:Chl-*a* ratio derived from MODIS-*Aqua* combined with a map of temperature-dependent nutrient limitation.

While data on long-term changes in the phytoplankton community exists, we do not have comparable time series of phytoplankton elemental stoichiometry. Using a trait-based phytoplankton model coupled with satellite inputs^38^, we estimated long-term patterns in the phytoplankton C:P ratio in the Sargasso Sea (Fig. 7). Over the available temporal record, 2003–2020, the modeled phytoplankton C:P ratio was similar in magnitude to the bulk seston C:P ratio (Fig. 6). The modeled phytoplankton C:P ratio significantly increased throughout the 2010s (Model 1 Regression, *P* = 0.002; slope = 4.3 ± 1.4 units year^−1^), which due to high variance, is statistically similar to the increase in the seston C:P ratio (*P* < 0.001; slope = 14.5 ± 2.9 units year^−1^). The change in modeled phytoplankton C:P is based upon using *Synechococcus* to represent the phytoplankton community, due to lack of validation datasets for other phytoplankton taxa, but this should also qualitatively reflect the change in the C:P *Prochlorococcus*^39^, which combined, constitute the largest portion of phytoplankton in the Sargasso Sea^40^. This increase in modeled phytoplankton C:P, for a taxonomically “constant” phytoplankton assemblage, is hypothesized to be due to slight decreases in growth rates that have a disproportionate impact on C:P ratios^38^. Neither the observational estimates of phytoplankton growth rate based upon carbon nor the modeled growth rate (Supplementary Fig. 9) significantly decreased through the 2010s, but all displayed a negative slope (Fig. 4D). This suggests that physiological adjustments that increased cellular C:P ratios had not yet resulted in significant decreases in growth rate.

While the temporal patterns of stoichiometric ratios in the euphotic zone mirrored those of exported particulate matter, C:P ratios in the euphotic zone were consistently and significantly lower (sample-to-sample Student’s *t* test; *P* < 0.001) than ratios at the 150-m sediment trap, suggesting enhanced shallow remineralization of both P and N between 100 and 150 m^41,42^. Most clearly, for the coupling of C:P ratios, it appears that both changes in the ratio of source material and enhanced remineralization of P are important. Based on the change over time in seston C:P ratios relative to changes over time in C:P ratios of exported material, we estimate ~20% of the change in the ratio of exported material is due to this change in the source material. By difference, ~80% of the change is due to enhanced shallow demineralization, which has been hypothesized as an important mechanism to sustain carbon export in oligotrophic regions^43^. Data for bacterial productivity between 100 and 150 m is scant during the decade of the 2010s, thus we cannot assess if the apparent increase in shallow remineralization is associated with changes in bacterial productivity, zooplankton “repackaging”^44^, and/or other unidentified processes.

Pico- and nanoeukaryotes in the Sargasso Sea are estimated to contribute ~10% of carbon export, which is at or below their contribution to carbon biomass—however, the precise mechanisms are poorly known^17^. Thus, while the loss of small eukaryotes should have had a detrimental impact on carbon export, it is conceivable that the resulting increase in the relative contribution of cyanobacteria with elevated C:P ratios maintained carbon export rates. Laboratory^45,46^ and field^47–49^ studies in the Sargasso Sea have confirmed that cyanobacteria are incorporated into large phytodetrital aggregates and fecal aggregates through physical interaction and “sticking”, thus potentially increasing their importance in carbon export. Phytodetrital aggregates alone have been shown to contribute 30–70% of measured bulk POC flux^47^. In contrast, studies have clearly shown that these picoplankton populations are tightly controlled by micrograzers^50–52^ and that “excess” carbon, relative to the needs of microzooplankton grazers, is respired during grazing^53^. Thus, this tight micrograzer control could counter an increased contribution of these picophytoplankton to carbon export.

Another mechanism for the lack of reduction in carbon export is an extensive but changing role of macrograzers as mediators of carbon flux; a hypothesis consistent with the high frequency of “zooplankton gut microbes” associated with fecal aggregates^47^. Salps are gelatinous filter feeders that can also effectively feed on picophytoplankton^54^ and they produce large, fast-sinking fecal pellets^55^. In contrast to crustacean zooplankton and microzooplankton, salps do not appear to respire excess carbon from their food leading to C:P ratios in their fecal material equal to or greater than suspended POM and the C:P ratio in exported material (Supplementary Fig. 8). Salp blooms occur periodically in the Sargasso Sea, dominating the zooplankton community and carbon export. An analysis of salp abundance at BATS from 1994 to 2011 indicates a long-term increase in the most common salp species^56,57^, which is positively correlated with a water column stratification index (analogous to our seasonal mixing depth amplitude). While the time series post-2011 has not been analyzed specifically for salps, we examined a metric that could reflect changes in salp abundance, zooplankton dry to wet weight ratio (in which an increase in salp abundance would result in a relatively lower ratio), however, no significant trends were observed. We also examined size-fractioned zooplankton biomass data to determine more broadly if there have been changes in community structure. Both day- and nighttime absolute zooplankton biomass of the largest (>5 mm) size class increased significantly in the period before 2010, and significantly decreased in the 2010s decade (Model 1 Regression, LN normalized data, *P* < 0.001) (only nighttime data shown, Supplementary Fig. 7a), however the fraction of zooplankton biomass >5 mm relative to total zooplankton biomass >0.2 mm did not change significantly before and after 2010 (Supplementary Fig. 7b), suggesting no change in the relative size structure of the zooplankton over the entire time series. At this time, we cannot sum these potential food web trophic changes with enough confidence to evaluate their contributions to maintaining elevated carbon export rates in the face of reduced phytoplankton biomass and mean population cell size.

We identify a counter-intuitive biogeochemical response as a result of warming in the subtropical Sargasso Sea. The 2010s saw warming faster than other decades in the past 30 years, depletion of euphotic zone nutrient inventories in response to more intense seasonal stratification, and the anticipated reduction in NPP. What was not observed is a coordinated reduction in carbon export, despite a relative increase in cyanobacteria and a decrease in total phytoplankton biomass, as the ecosystem improved its P use efficiency. These results suggest changes in the phytoplankton community and trophic interactions controlling the continued export of carbon and remineralization of limiting nutrients are not adequately captured in current earth system models.

## Methods

### Study location and overview

The history of the Sargasso Sea ocean time-series research and basic understanding of the physical and biological characteristics of this region are described in detail in prior reviews^24,58,59^. On each monthly (biweekly in the spring) BATS cruise, a quasi-lagrangian sampling scheme is employed, and for this manuscript, all CTD/hydro casts falling within a 0.25° latitude by 0.25° longitude box centered around the BATS site (31°40’N; 64°10’W) are presented. Further details of the sampling scheme, analytical methods, data quality control (QC) and quality assurance (QA), and history of sampling procedures are available in the BATS methods manuals^60^ and in published papers^61–64^. Detailed methods are provided below.

### Hydrography

Continuous CTD data were collected from the downcast and calibrated against discrete samples (e.g., ref. ^24^). CTD profile data were used to estimate mixed layer depths using a 0.2 °C variable sigma-θ criterion^65^. Near-surface (10 m) temperature anomalies were generated by seasonally detrending the entire data record.

### Dissolved inorganic, organic, and particulate nutrients

Samples for nitrate and phosphate were filtered (0.8-µm polycarbonate filter) into acid-washed HDPE bottles, and frozen (−20 °C) until analysis using standard air-segmented autoanalyzer methods^66^. In addition, starting in October 2004, samples were collected for high-sensitivity phosphate analyses using the Magnesium Induced Co-precipitation (MAGIC) soluble reactive phosphorus (SRP) method^67,68^, as modified for BATS^31^. During each sample run, commercially available certified standards, OSIL and Wako Chemical, are analyzed to maintain consistent data quality, as well as “standard water” from 3000 m that serves as an internal standard.

Particulate organic carbon (POC), nitrogen (PON), and phosphorus (PP) samples were filtered on precombusted (450 °C, 4 h) Whatman GF/F filters and frozen until analysis on a Control Equipment 440-XA elemental analyzer^59^. Particulate phosphorus samples (PP) were analyzed using an ash-hydrolysis method with oxidation efficiency and standard recovery checks^31^.

### Picoplankton abundance and biomass

Samples for pico- and nanoplankton enumeration were collected on each cruise from June 2002 to the present^69^. Samples were preserved (0.5% v/v paraformaldehyde) and flash-frozen in liquid nitrogen before being stored at −80 °C until analysis by flow cytometry. Small cyanobacteria were identified as either *Synechococcus* or *Prochlorococcus* based upon cell size and the presence or absence of phycoerythrin, respectively. Eukaryotes were defined as other chlorophyll-containing cells not being cyanobacteria. They were separated into picoeukaryotes (<3 µm) and nanoeukaryotes (>3 µm) based upon their forward scatter signal relative to 3-µm polystyrene beads. Phytoplankton cell abundance was converted to carbon per cell using a normalized cell size-carbon relationship and then to population biomass by multiplying by cell abundance (^40^).

Total phytoplankton carbon was estimated independently by regressing integrated (0–140 m) total POC against total chlorophyll (Chl-*a*) to obtain a single slope for the dataset that relates the two parameters. This approach was used rather than taking the average ratio of discrete POC and Chl-*a* integrals as it allows for the exclusion of “non-phytoplankton” POC (i.e., the *y* intercept in the regression) from the relationship between phytoplankton POC and Chl-*a*. The slope of the POC:Chl-*a* regression was then multiplied by integrated Chl-*a* to estimate phytoplankton POC. This estimate of total phytoplankton carbon was compared to the sum of flow cytometry-derived picoplankton carbon, with any differences indicative of larger microplankton not well measured by flow cytometry.

### Particulate elemental fluxes

Sinking fluxes of POC, PON, PP from the euphotic zone were quantified using surface-tethered particle interceptor traps^24,59,70^. Fluxes were calculated from the elemental mass of material captured in the sediment trap, scaled to the sediment trap tube surface area and array deployment duration. A filter blank correction, based on three blank particle interceptor traps, was applied to POC and PON fluxes starting in October 2008. Paired analysis of “raw” and “blank-corrected” flux rates show that the blank correction was 2.3% of raw POC fluxes and 4.5% of PON fluxes across the entire range of fluxes. These blank corrections were applied uniformly to the entire data record to provide consistency throughout the whole data record.

### Primary production

In situ production estimates were determined using surface-tethered arrays with samples spaced every 20 m between the surface and 140 m. Rates of primary production were calculated from the incorporation of H^14^CO_3_^−^ into particles (i.e., particles nominally >0.7 µm) using an assumed ratio of total inorganic carbon present to radiocarbon added. From 1990 through 2005, samples were collected with Go-Flo bottles on a Kevlar line, and from November 2004 to the present, samples were collected from Niskin bottles on the CTD rosette. Rates of primary production were corrected for dark carbon uptake and integrated to a depth of 140 m^23^.

### Trait-based model

The trait-based phytoplankton stoichiometry model is a modification of Inomura et al.^71^, which facilitates the accurate computation of phytoplankton C:N:P under a variety of environmental conditions. Here, the original trait-based based model was modified following Tanioka et al.^38^ to predict phytoplankton C:P as a function of satellite-based growth rate, Chl:C, and P limitation. We used the model parameter set for the freshwater cyanobacteria ﻿*Synechococcus linearis* except for a hard-bound maximum C:P of 335 at the zero growth rate, following experimental chemostat data on the marine cyanobacteria *Synechococcus* WH8102^72^. With this slight modification to the model parameters, the model C:P matches the observed C:P from the culture experiment when the growth rate is less than 0.8 d^−1^ (Supplementary Fig. 7). The model overestimates the observed C:P by ~40 at the highest growth rate, possibly due to the simplified nature of the storage pool representation. However, as the typical seasonal maximum growth rate observed at BATS rarely exceeds 0.8 day^−1^ (Fig. 4C), the model can accurately predict satellite-driven C:P.

We report here a monthly and area-averaged C:P for a 3-by-3 pixel around BATS predicted using the trait-based model forced with satellite input derived from MODIS-aqua. Satellite-derived monthly growth rate and phytoplankton carbon (C_phyto_) are based on the estimates from the Carbon-based Productivity Model (C_bPM_)^73^. Chl:Cphyto ratio, a proxy for light limitation, is computed by dividing MODIS-derived Chl-*a* with C_phyto_. Both growth rate and Chl:C_phyto_ are assumed to be vertically uniform in the mixed layer. P limitation is inferred by comparing MODIS-derived monthly mean SST and empirically derived phosphate depletion temperature, a temperature above which phosphate is no longer detectable^74^.

## Supplementary information

Supplementary Information

## Data Availability

All observational data for the BATS time series can be found either on the BATS data server (http://bats.bios.edu/bats-data/) or on the BATS project page at the Biological-Chemical Oceanography Data Management Office (https://www.bco-dmo.org/project/2124). Output from the trait-based model, and its code, can be reasonably requested directly from co-author Tatsuro Tanioka.
