# Supplementary material for: Adaptive carbon export response to warming in the Sargasso Sea
Source: Nat Commun. 2022 Mar 8;13:1211. doi: 10.1038/s41467-022-28842-3 (PMC8904855; doi:10.1038/s41467-022-28842-3)
Supplement: Supplementary file 1 — Supplementary Information [file 41467_2022_28842_MOESM1_ESM.pdf]

Supplementary Information:

Adaptive Carbon Export Response to Warming in the Sargasso Sea.

Michael W. Lomas[1], Nicholas R. Bates[2,3], Rodney J. Johnson[2], and Deborah K. Steinberg[4], Tatsuro Tanioka[5],

[1] Bigelow Laboratory for Ocean Sciences, East Boothbay, ME, USA

[2] Bermuda Institute for Ocean Sciences, St. Georges, Bermuda

[3] Department of Ocean and Earth Science, University of Southampton, Southampton, UK

[4] Virginia Institute of Marine Science, William & Mary, Gloucester Pt., VA, USA

[5] Department of Earth System Science, University of California, Irvine, CA, USA

Keywords: elemental stoichiometry, climate change, phytoplankton community, carbon export, primary production

Target Journal: Nature Communications

Corresponding Author: Michael W. Lomas, mlomas@bigelow.org

Additional Methodological Details and Results.

North Atlantic Oscillation (NAO) Index. The NAO, specifically the winter DJFM NAO index value, has been shown to be a variable that correlates with a range of hydrographic, chemical, and biological variables. Data from Hurrell and colleagues [1] are plotted below, showing the pattern of DJFM NAO values for the duration of the BATS time series (**Supplementary Figure 1**). There are two winters with substantial negative NAO index values, 1996 and 2010, with the wintertime NAO negative phase in 2010 being of particular note [2].

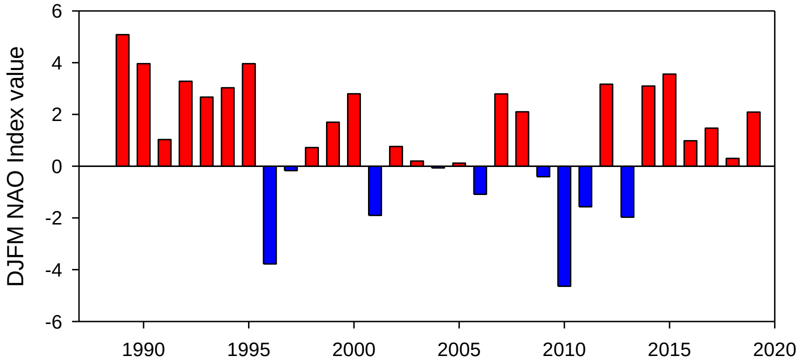

Supplementary Figure 1. Winter (DJFM) NAO index values for the duration of the BATS time series. Data compiled by Hurrell and colleagues [1].

Dissolved Organic Phosphorus (DOP). The DOP pool is the dominant phosphorus pool in the Sargasso Sea [3,4] and is potentially utilized to support primary production; particularly during the winter/spring bloom. Samples for total phosphorus have been collected since 2004. Samples were collected into acid-washed HDPE bottles and frozen (-20°C) until analysis. Total phosphorus concentrations are quantified using a combined high temperature/persulfate oxidation – MAGIC sample concentration technique [4]. DOP concentrations were calculated by subtracting inorganic phosphate and particulate phosphorus concentrations from total phosphorus concentration. Sample accuracy was checked by analysis of a certified standard (OSIL Phosphate Nutrient Standard Solution) with each sample run.

The integrated DOP pool did not significantly change, increase or decrease, from 2004-2010 (**Supplementary Figure 2**). Throughout the 2010's decade, DOP inventories decreased significantly (Model 1 Linear Regression, $R^2 = 0.10$; slope = -0.254 ±0.057 mmol m^{-2} y^{-1}; $P < 0.001$. Rate of decline in inventory is 2-4% y^{-1}).

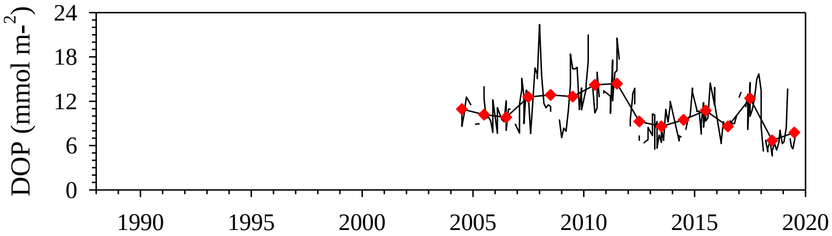

Supplementary Figure 2. Time-series of euphotic zone integrated dissolved organic phosphorus (DOP) inventory. Red diamonds represent annual mean values.

Elemental Particle Fluxes. Surface-tethered sediment traps at the Bermuda Atlantic Time-series Study (BATS) site are deployed at three depths, 150 m, 200 m, and 300 m. The data for the 200 m and 300 m traps depths are present here (**Supplementary Figure 3, 4**). These depths show the same general patterns as the 150 m trap (**Figure 3** in primary text); C:N ratios did not exhibit a significant trend over the entire record, whereas C:P (Model 1 regression, P <0.001, slope:200m = 105 ± 22 units y^{-1}; slope:300m = 112 ± 21 units y^{-1}) and N:P (Model 1 regression, P <0.001, slope:200m = 8.1 ± 2.0 units y^{-1}; slope:300m = 15.0 ± 3.8 units y^{-1}) ratios both increased significantly throughout the 2010's, relative to the prior decades. The sharp increase in C:P and N:P ratios was consistent with the temporal pattern observed in the 150m trap, suggesting a broader system response. Between each depth pair (i.e., 150-200 m, 200-300 m), C:P and N:P ratios significantly increased due to the continual selective remineralization of phosphorus [5]. C:N ratios, on the other hand, are only significantly different between 150-200 m.

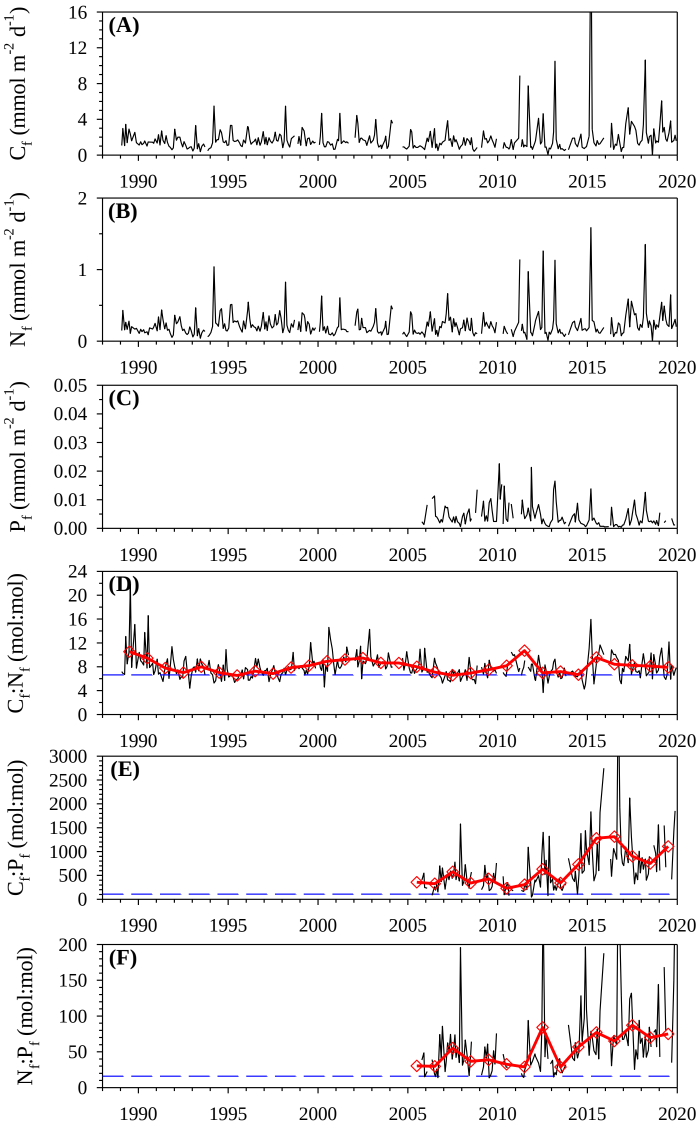

Supplementary Figure 3. Time-series of elemental export fluxes and stoichiometric ratios at 200m. (**A**) Particulate organic carbon (C_f) flux; (**B**) Particulate organic nitrogen (N_f) flux; (**C**) Particulate phosphorus (P_f) flux; (**D**) particulate C_f:N_f flux ratio; (**E**) particulate C_f:P_f flux ratio; and (**F**) particulate N_f:P_f flux ratio. In panels D-F, the red triangles represent the annual mean flux ratio, and the blue dashed line represents the Redfield Ratio.

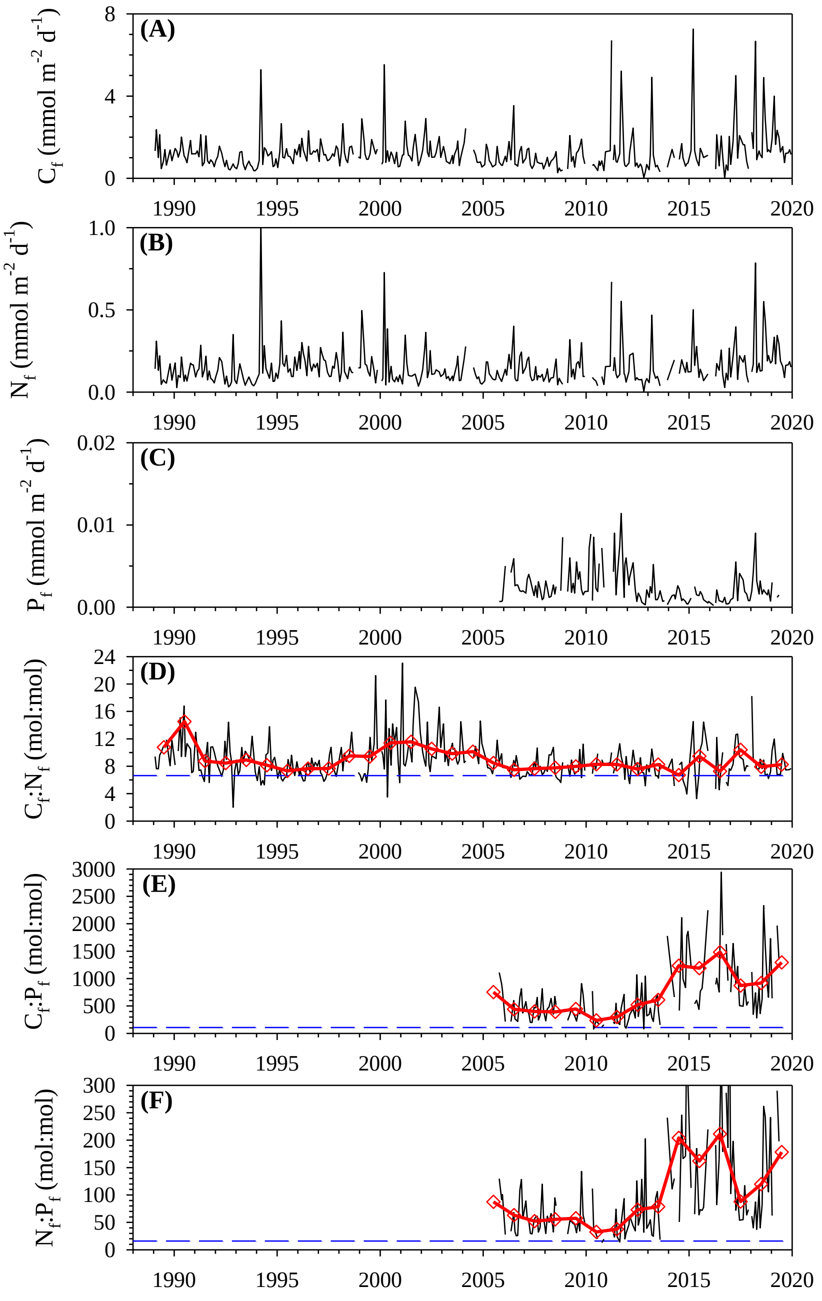

Supplementary Figure 4. Time-series of elemental fluxes and stoichiometric ratios at 300 m. (**A**) Particulate organic carbon (C_f) flux; (**B**) Particulate organic nitrogen (N_f) flux; (**C**) Particulate phosphorus (P_f) flux; (**D**) particulate C_f:N_f flux ratio; (**E**) particulate C_f:P_f flux ratio; and (**F**) particulate N_f:P_f flux ratio. In panels D-F, the red triangles represent the annual mean flux ratio, and the blue dashed line represents the Redfield Ratio.

Seston Stoichiometry. Stoichiometry of seston particulate organic matter at BATS is highly correlated between the upper and lower euphotic zone (r>0.67 for all ratios). However, the

stoichiometric ratios differ in their magnitude between the upper and lower euphotic zones. C:P and N:P ratios are lower in magnitude in the lower euphotic zone, consistent with the notion that vertical nutrient inputs are important and that phytoplankton in these lower depths assimilate the upward flux of nutrients resulting in a less nutrient stressed condition than their counterparts in the upper euphotic zone. Seston stoichiometric ratios for the lower euphotic are given in the main manuscript (**Figure 4** in the primary text), and the upper euphotic zone stoichiometric ratios are presented here (**Supplementary Figure 5**). C:P ratios in the lower euphotic zone increased significantly throughout the 2010's (Model 1 regression, P <0.001, slope = 14.6 ± 3.0 units y^{-1}), but not during the years before. N:P ratios in the lower euphotic zone increased marginally (Model 1 regression, P = 0.09, slope = 0.8 ± 0.5 units y^{-1}), but not during the years before.

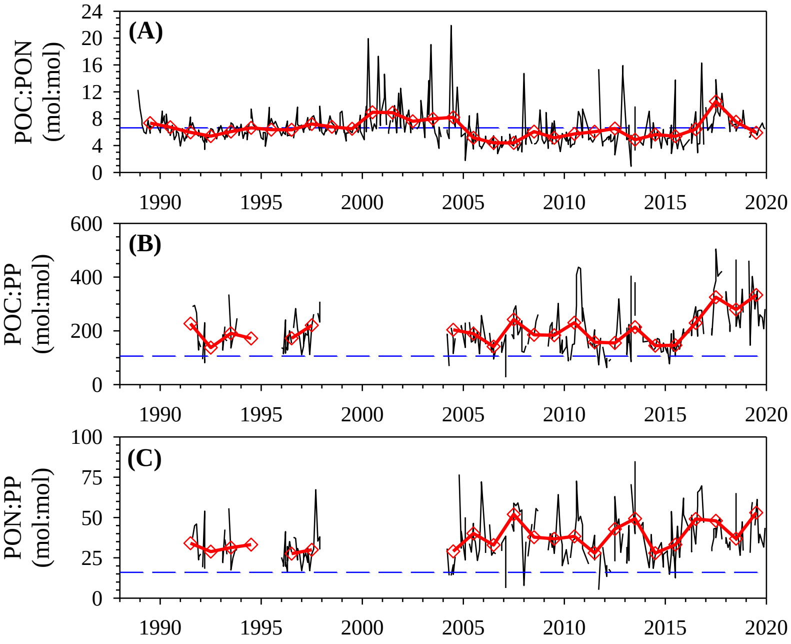

Supplementary Figure 5. Time-series of seston elemental stoichiometric ratios in the lower (60-100 m) euphotic zone layer. (**A**) POC:PON; (**B**) POC:PP; (**C**) PON:PP. Red triangles represent the annual mean stoichiometric ratio, and the blue dashed line represents the Redfield Ratio.

Living Phytoplankton Carbon. We assessed the contribution of phytoplankton carbon to total POC stocks, by calculating the ratio of our flow cytometry phytoplankton carbon estimate (FCM PhytoC) to total POC. Quantification of total phytoplankton POC is described in the summary methods in the primary text. Contributions of phytoplankton POC ranged from 0.11-0.71 and averaged 0.30 ± 0.11. There was no significant change in the contribution before or after 2010.

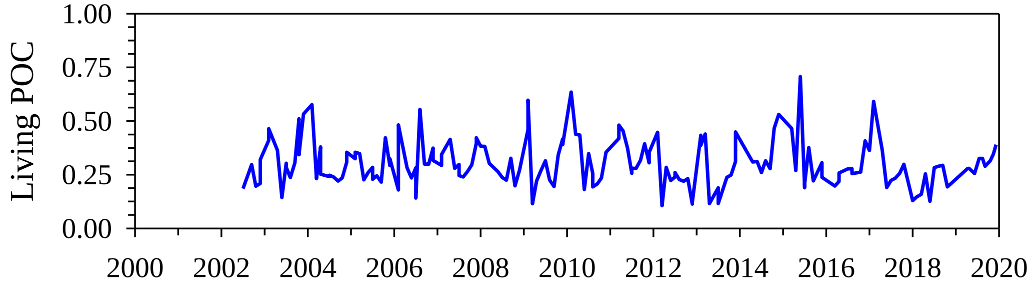

Supplementary Figure 6. Phytoplankton carbon as a proportion of total suspended POC.

Zooplankton biomass, fecal pellets, and stoichiometry

Samples for zooplankton biomass were collected using vertically integrated (0 m to a targeted maximum depth between 150–200 m) double oblique tows conducted during the day (0900 to 1500) and night (2000 to 0200)(only night data are presented here) with a rectangular frame (0.8 x 1.2 m) net with 202 µm mesh [6,7]. Samples were size-fractionated through sieves, 0.2-0.5mm, 0.5-1mm, 1-2mm, 2-5mm, and >5mm, washed onto smaller Nitex filters, rinsed with Milli-Q water to remove salt, and dried to constant weight [8]. Absolute zooplankton biomass in the >5mm fraction, natural log-transformed, increased significantly before 2010 (Model 1 regression, $P < 0.001$, slope = 1.06 ± 1.01 mg DW m^{-3}), but decreased after 2010 (Model 1 regression, $P < 0.001$, slope = -0.91 ± 1.02 mg DW m^{-3}) (**Supplementary Figure 7a**). In contrast, the relative biomass (>5mm biomass as a fraction of total biomass >0.2mm) did not change significantly (**Supplementary Figure 7b**).

During summer cruises conducted as part of the Trophic BATS program in 2011 and 2012, crustacean zooplankton bodies and zooplankton and salp faecal pellets produced during onboard incubations were provided by Dr. Stephanie Wilson and analyzed for their C, N, and P content. The POC:POP ratio in salp faecal pellets was substantially greater than the POC:POP ratio in suspended particulate matter and was more similar to the POC:POP ratio in POM captured in 150 m surfaced tethered traps from these cruises (**Supplementary Figure 8**). This is in contrast to the crustacean zooplankton, which had a POC:POP ratio in their body tissue lower than the averaged POC:POP ratio in suspended matter and produced fecal pellets that were even more enriched in P than body tissue.

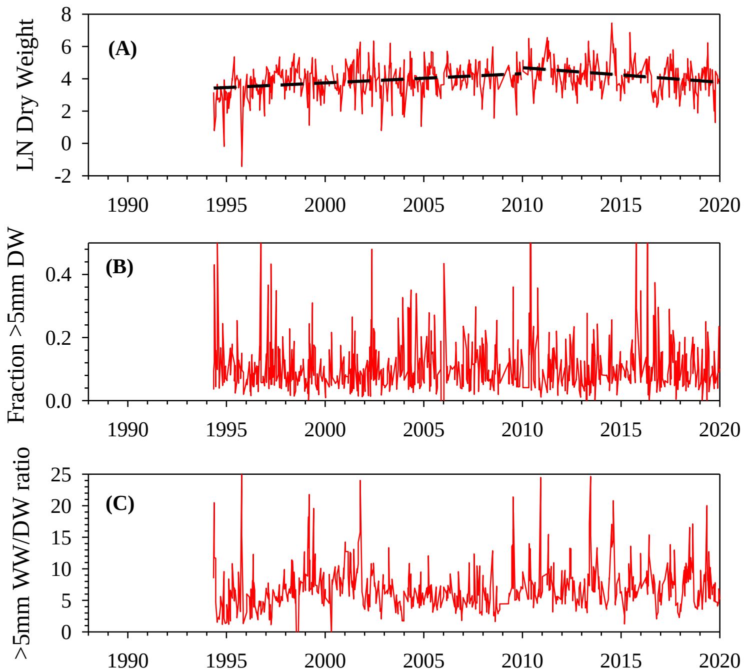

Supplementary Figure 7. Nighttime zooplankton biomass in the >5mm size fraction. (A) natural log (LN) transformed absolute biomass (mg DW m^{-3}), (B) >5mm biomass relative to the total zooplankton biomass >0.2mm and (C) wet weight to dry weight ratio in the >5 mm size fraction. Heavy dashed lines in (A) are the regression lines showing the increase and the decrease before and after 2010.

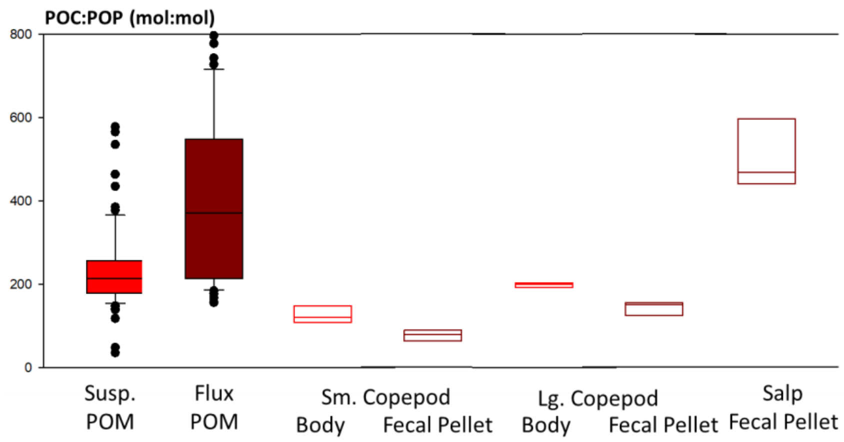

Supplementary Figure 8. POC:POP ratios in suspended organic matter (Susp. POM), sediment trap organic matter (flux POM), small (<2 mm) and large (>2 mm) copepod body tissue and fecal pellets, and salp fecal pellets collected during summer cruises in the Sargasso Sea. Data are presented as box-whisker plots. The box represents the 25th and 75th quartile, the line in the box represents the median, the error bars represent the 5th and 95th percentiles, and solid data points represent values outside the 5th and 95th percentiles. For the copepod and salp body tissue and fecal pellets, there are limited data points and thus only the 25th and 75th quartiles and the median are presented.

Trait-based model. The phytoplankton stoichiometry model of Inomura et al. [9] is a conceptually simple mechanistic model that facilitates the accurate computation of phytoplankton C:N:P under different environmental conditions. The input variables are light intensity, growth rate, and the presence/absence of limiting nutrients. The model is based on four empirically supported lines of evidence: (1) a saturating relationship between light intensity and photosynthesis, (2) a linear relationship between the RNA-to-Protein ratio and growth rate, (3) a linear relationship between biosynthetic protein and growth rate, and (4) a constant macromolecular composition of the light-harvesting machinery.

Inomura et al. [9] calibrated the model parameters subject to constraints from published chemostat studies for multiple key marine and freshwater phytoplankton species. We used the model parameter set for the cyanobacteria *Synechococcus linearis* because cyanobacteria such as *Synechococcus* and *Prochlorococcus* are the most abundant phytoplankton types at BATS. However, as this species of *Synechococcus* is a freshwater species, we separately obtained the parameter related to maximum C:P at the zero-growth rate from a chemostat experiment for marine *Synechococcus* species WH8102 ([10]). **Supplementary Figure 9** shows that the trait-based model accurately captures the C:P of P-limited *Synechococcus* at different growth rates as long as the growth rate is less than 0.8 d^{-1}. As the typical seasonal maximum growth rate observed at BATS rarely exceeds 0.8 d^{-1} (**Figure 4D**), we believe that this will not undermine the model's ability to predict phytoplankton C:P ratios at BATS.

The modeled growth rate estimates did not show a significant trend before or after 2010, and rates in the two periods were not statistically different. We compared the modeled growth rate to growth rate estimates based upon direct observational measurements (**Figure 4D** in the primary text). Modeled growth rates were not significantly correlated (Pearson Product Moment Correlation) with either observational growth rate estimate, although the two observational estimates were well correlated with each other (**Supplementary Table 1**). The modeled growth rate (mean ± Std. Err.: 0.52 ± 0.014 d^{-1}) was significantly greater (one-way ANOVA, $P<0.05$) than both the flow cytometry carbon-based growth rate (0.38 ± 0.015 d^{-1}) and the C:Chlorophyll-based growth rate estimate (0.46 ± 0.14 d^{-1}). The likely reason for the modeled growth rates being higher than the observation-based growth rates is that the model was developed for *Synechococcus*, while the observations include carbon associated with a wide diversity of eukaryotic phytoplankton.

Supplementary Table 1. Pearson Product Moment Correlations of the three methods for estimating phytoplankton growth rates. In each cell, Row 1 = correlation coefficient r, Row 2 – P-value, and Row 3 – number of data points in the correlation.

	FCM growth rate	Model growth rate
C:Chl growth rate	0.55<br><0.001<br>133	-0.09<br>0.27<br>155
FCM growth rate		-0.08<br>0.365<br>117

Following Tanioka et al. [11], we drove the Inomura model with satellite-derived phytoplankton growth rate, Chl:C ratio of phytoplankton biomass, and P limitation [as the difference between satellite-derived SST and cubit root-corrected phosphate depletion temperature (PDT3)] to estimate phytoplankton C:P in the surface layer. In doing so, we made three modifications to the original stoichiometry model by Inomura et al. [9] following the protocol by Tanioka et al. [11]. First, we used depth-integrated POC:Chl-a from the satellite ocean color instead of calculating POC:Chl-a as a function of photon-flux density. Second, we imposed a fixed maximum growth rate of 2 d^{-1} in calculating C:P, which is equal to the maximum growth rate imposed on the satellite-based estimates of growth rate [12]. Third, we imposed a constant C:P value of 102 under the P-replete condition regardless of the P supply.

We used monthly and area-averaged satellite (MODIS *Aqua*) derived variables for a 3-by-3 pixel area around BATS to predict phytoplankton C:P in the top 100 m between 2003 and 2020. All satellite-derived input data and estimates of mixed-layer depth are available for download from the Oregon State Ocean Productivity Website [http://sites.science.oregonstate.edu/ocean.productivity/index.php (last access: June 21, 2021)]. The original phytoplankton stoichiometry model codes are publicly available (http://doi.org/10.5281/zenodo.4414338), and the modified version used in this study is available upon request.

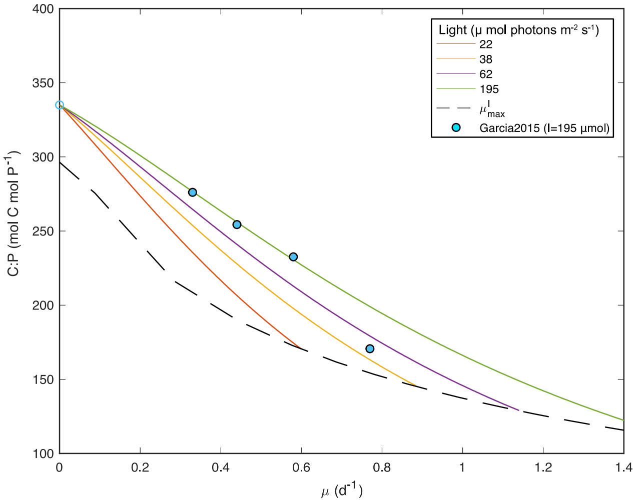

Supplementary Figure 9. Model-data comparison of C:P of *Synechococcus* for various growth rates. Curves: Trait-based model C:P under different light intensities (μmol m^{-2} s^{-1}). Circles: laboratory data for C:P of *Synechococcus* WH8102 grown under P-limited chemostat culture at a photon flux density of 195 mol m^{-2} s^{-1} (Garcia et al. 2016). The open blue circle is the experimentally derived maximum C:P of 335 at the zero-growth rate. The dashed line represents C:P at maximum growth rate (μ^{I}_{max}) at various light intensities; high μ^{I}_{max} for higher light intensity.

Data Availability Statement: All observational data for the BATS time-series can be found either on the BATS data server (http://bats.bios.edu/bats-data/) or on the BATS project page at the Biological-Chemical Oceanography Data Management Office (https://www.bco-dmo.org/project/2124). Output from the trait-based model, and its code, can be reasonably requested directly from co-author Tatsuro Tanioka.

Acknowledgements: The authors acknowledge and thank the numerous principal investigators, researchers, and technicians who have contributed to the BATS time-series project since its inception. Additional thanks go to the officers and crew of the R/V Weatherbird I, R/V Weatherbird II and R/V Atlantic Explorer. NSF funding is acknowledged for MWL (OCE-1756054), DKS (OCE-1756312) and NRB and RJJ (OCE-1756105).

Author Contributions Statement: MWL conducted the analyses and wrote the first draft of the manuscript. RJJ assisted with providing data. TT contributed the results of and text describing the phytoplankton C:P model. DKS, NRB, RJJ and TT edited initial and revised drafts of the manuscript.

Competing Interests Statement: All authors declare no competing interests.

Supplementary References:

1. Hurrell, J. *The Climate Data Guide: Hurrell North Atlantic Oscillation (NAO) Index (station-based)*, < https://climatedataguide.ucar.edu/climate-data/hurrell-north-atlantic-oscillation-nao-index-station-based> (2020).
2. Osborn, T. J. Winter 2009/2010 temperatures and a record-breaking North Atlantic Oscillation index. *Weather*, https://doi.org/10.1002/wea.1660 (2010).
3. Ammerman, J. W., Hood, R. R., Case, D. & Cotner, J. B. Phosphorus deficiency in the Atlantic: an emerging paradigm in oceanography. *EOS* **84**, 165-170 (2003).
4. Lomas, M. W. *et al.* Sargasso Sea phosphorus biogeochemistry: an important role for dissolved organic phosphorus (DOP). *Biogeosciences* **7**, 695-710 (2010).
5. Tanioka, T., Matsumoto, K. & Lomas, M. W. Drawdown of Atmospheric pCO2 via Dynamic Particle Export Stoichiometry in the Ocean Twilight Zone. *Earth and Space Science Open Archive*, https://doi.org/10.1002/essoar.10507405.10507401 (2021).
6. Madin, L. P., Horgan, E. F. & Steinberg, D. K. Zooplankton at the Bermuda Atlantic Time-series Study (BATS) station: diel, seasonal and interannual variation in biomass, 1994-1998. *Deep-Sea Research II* **48**, 2063-2082 (2001).
7. Steinberg, D., Lomas, M. & Cope, J. Long-term increase in mesozooplankton biomass in the Sargasso Sea: Linkage to climate and implications for food web dynamics and biogeochemical cycling *Glob. Biogeochem. Cycle* (2012).
8. Lomas, M. W. *et al.* Two decades and counting: 24-years of sustained open ocean biogeochemical measurements in the Sargasso Sea. *Deep Sea Research II* **93**, 16-32 (2013).
9. Inomura, K. *et al.* A mechanistic model of macromolecular allocation, elemental stoichiometry, and growth rate in phytoplankton. *Frontiers in Microbiology* **11**, doi: 10.3389/fmicb.2020.00086 (2020).
10. Garcia, N., Bonachela, J. A. & Martiny, A. C. Interactions between growth-dependent changes in cell size, nutrient supply and cellular elemental stoichiometry of marine Synechococcus. *ISME Journal* **10**, 2715-2724 (2016).
11. Tanioka, T., Fichot, C. & Matsumoto, K. Toward determining the spatio-temporal variability of upper-ocean ecosystem stoichiometry from satellite remote sensing. *Frontiers in Marine Science* **7**, 604893. doi: 604810.603389/fmars.602020.604893 (2020).
12. Westberry, T. K., Behrenfeld, M. J., Siegel, D. A. & Boss, E. Carbon-based primary productivity modeling with vertically resolved photoacclimation. *Glob. Biogeochem. Cycle* **22**, GB2024, doi:2010.1029/2007GB003078 (2008).
